# Lifespan outperforms climate as a predictor of wood functional traits, but secondary woodiness shows no clear climatic pattern in *Heliophila*, a diverse clade from the Cape Floristic Region

**DOI:** 10.1093/aob/mcaf046

**Published:** 2025-03-19

**Authors:** J Baczyński, A A Oskolski, P J D Winter, R Manuel, T Lyner, A R Magee, A M Muasya, K E Frankiewicz

**Affiliations:** Institute of Evolutionary Biology, Faculty of Biology, Biological and Chemical Research Centre, University of Warsaw, Żwirki i Wigury 101, 02-089 Warsaw, Poland; Department of Plant Biology, Miller Plant Sciences, University of Georgia, 120 Carlton Street, Athens, GA, USA; Department of Botany and Plant Biotechnology, University of Johannesburg, PO Box 524, Aukland Park 2006, Johannesburg, South Africa; Komarov Botanical Institute, Prof. Popov Street 2, 197376, St. Petersburg, Russia; Compton Herbarium, South African National Biodiversity Institute, Private Bag X7, Claremont 7735, Cape Town, South Africa; Compton Herbarium, South African National Biodiversity Institute, Private Bag X7, Claremont 7735, Cape Town, South Africa; Compton Herbarium, South African National Biodiversity Institute, Private Bag X7, Claremont 7735, Cape Town, South Africa; Department of Botany and Plant Biotechnology, University of Johannesburg, PO Box 524, Aukland Park 2006, Johannesburg, South Africa; Compton Herbarium, South African National Biodiversity Institute, Private Bag X7, Claremont 7735, Cape Town, South Africa; Bolus Herbarium, Department of Biological Sciences, Faculty of Science, University of Cape Town, Private Bag X3, Rondebosch 7700, South Africa; Compton Herbarium, South African National Biodiversity Institute, Private Bag X7, Claremont 7735, Cape Town, South Africa; Bolus Herbarium, Department of Biological Sciences, Faculty of Science, University of Cape Town, Private Bag X3, Rondebosch 7700, South Africa; Plant Cytogenetics and Molecular Biology Group, Institute of Biology, Biotechnology and Environmental Protection, Faculty of Natural Sciences, University of Silesia in Katowice, 40-032 Katowice, Poland

**Keywords:** Annual, Brassicaceae, Cape Floristic Region, climate response, functional traits, *Heliophila*, lifespan shifts, perennial, phylogenetic inertia, secondary woodiness, wood anatomy, wood evolution

## Abstract

**Background and Aims:**

Annuals produce little wood due to their short life cycle, while perennials can accumulate more, though not all do. Consequently, lifespan extension is a prerequisite for – but not synonymous with – secondary woodiness. Even if a shift to perenniality does not substantially increase wood production, it may still affect wood anatomy, as annuals prioritize rapid growth, whereas perennials invest in structural resilience. *Heliophila*, a genus of the Brassicaceae from the Cape Floristic Region, provides an excellent system to investigate drivers of secondary woodiness and the impact of lifespan shifts on wood traits due to its multiple independent lifespan transitions and occurrence of secondary woodiness.

**Methods:**

We reconstructed evolutionary transitions between annual and perennial lifespans and between herbaceous and secondarily woody habits. Using phylogenetically informed statistics, we analysed the relationship between climate, lifespan and nine wood anatomical traits. Lifespan-specific evolutionary optima for these traits were estimated and compared. We also tested whether secondary woodiness in *Heliophila* is associated with specific climatic niches.

**Key Results:**

Lifespan shifts in *Heliophila* are driven primarily by water availability and seasonality, with perennials evolving in wetter and less seasonal environments. Secondary woodiness may be more frequent in warmer niches, though this trend was not statistically supported, probably due to the limited number of secondarily woody species. Lifespan, not climate, better predicted wood traits: annuals had longer, thinner-walled cells, while perennials had shorter cells with thicker walls.

**Conclusions:**

In *Heliophila*, a shift in climatic niche prompts a change in lifespan, followed by slower adaptations in wood anatomy. Possibly, this pattern arises because alterations in lifespan affect stem architecture, establishing a developmental framework that governs subsequent anatomical adjustments. Furthermore, although not statistically robust, increased wood production may be linked to warmer niches, potentially associated with a temperature-driven enhancement in lignin biosynthesis that reinforces stem structure.

## INTRODUCTION

Plants adapt to seasonal drought through tolerance and avoidance, opposite extremes of the adaptive spectrum. Drought tolerance involves the development of traits associated with greater embolism resistance, whereas avoidance may result in the shortening of lifecycles, leading to plants surviving unfavourable periods in the form of seeds. A prolonged dry season increases the probability of adult mortality, and according to a classical theory, when adult mortality is high, short-lived annuals are selected over perennials (Schaffer and Rosenzweig, 1977; Stearns, 1998; Friedman, 2020). Consequently, drought-responsive traits experience different selective pressures in annual and perennial species.

Specifically, perennials are expected to be selected for traits that convey drought resistance and mechanical strength because their stems have to stay upright despite periodical turgor loss and remain functional over sequent growing seasons. In contrast, annuals are likely to be selected for traits conducive to rapid growth, allowing for the completion of the lifecycle during brief favourable periods. Wood (secondary xylem) is a convenient model for evaluating the distinctive pressures of both lifespans because mechanistic understanding and statistical correlations that explain strength and drought resistance are well documented (Lens *et al.*, 2022) (Table 1).

**Table 1.** Wood traits and their functional significance.

Wood trait(s)	Significance	References
Fibre and vessel wall thickness	Thicker walls in fibres and vessels enhance mechanical strength and increase wood density, crucial for maintaining structural integrity and contributing to the plant’s durability and longevity.	Carlquist, 2001; Kallarackal and Ramírez, 2024
Fibre and vessel element length	Both cell types originate from the same fusiform cambial initials, so while changes in the length of one cell type may be adaptive, the other may follow passively. Longer fibres may increase mechanical strength by enhancing cell contact, while shorter vessel elements may resist buckling.	Carlquist, 2001; Echeverría *et al.*, 2023
Vessel diameter, density, grouping	Wider vessels improve water transport efficiency but are more susceptible to cavitation and embolism. Narrower vessels reduce this risk, enhancing hydraulic safety. Higher vessel grouping offers alternative pathways if some vessels become embolized, improving hydraulic safety. Increased vessel density boosts water transport capacity but may result in smaller vessels.	Lens *et al.*, 2022; Olson *et al.*, 2023
Pit diameter	Pit diameter (measured in horizontal and vertical directions) could affect water movement between vessels and play a key role in preventing embolism – larger pits enhancing water flow and increasing the risk of air entry during drought. However, the significance of pit diameter is probably less than the characteristics of the pit membrane.	Lens *et al.*, 2013; Li *et al.*, 2019; Brodersen, 2021

Against a common misconception, non-monocot flowering plants generally produce a small amount of secondary xylem at the base of their stems (Schweingruber and Büntgen, 2013). Comparisons of secondary xylem from such plants with their woodier relatives have been conducted previously, with some involving an experimental approach (e.g. Lens *et al.*, 2016; Dória *et al.*, 2018, 2019), and others being more descriptive (e.g. Lens *et al.*, 2012; Schweingruber *et al.*, 2014; Kidner *et al.*, 2015; Frankiewicz *et al.*, 2022). None, however, modelled whether lifespan affects the selective pressures experienced by secondary xylem. Descriptive comparisons of wood anatomy between annual and perennial species may be uninformative in this respect, as they represent only a ‘snapshot’ of the current state of traits but not evolutionary trends. For instance, if a given species shifted to the annual lifespan in the recent evolutionary past, its anatomy may still resemble that of its perennial relatives while already being subject to the same selective pressures as other annuals in the same ecological niche. Evolutionary transitions between lifespans are among the most common transitions in angiosperms (Friedman and Rubin, 2015), so the problem of wood anatomy ‘lagging behind’ the lifespan may also be common.

Lifespan shifts can influence not only wood anatomy but also the amount of wood produced by a species. While annuals, due to their short lifespan, inherently produce little wood, perennials have the potential to accumulate more. What prompts perennials to realize this potential remains an open question – some may still produce only small amounts of wood, remaining in the ‘herbaceous’ category despite having undergone lifespan extension. Species that have evolved woodiness from herbaceous ancestors (defined as those that produce only a small amount of wood, often restricted to the stem base) are referred to as secondarily woody. Several hypotheses explain the selective pressures driving the evolution of secondary woodiness, ranging from competition for light and pollinators to climatic factors. Among climate-based explanations, the moderate climate hypothesis suggests that secondary woodiness evolves in response to a mild, seasonally stable climate (Carlquist, 1974, 2013). In contrast, secondary woodiness may also evolve in climates with seasonal drought (Lens *et al.*, 2013*a*, *b*; Zizka *et al.*, 2022). While these hypotheses offer different perspectives, they are not mutually exclusive – different clades may evolve woodiness in response to distinct selective pressures.

**Fig. 1.**
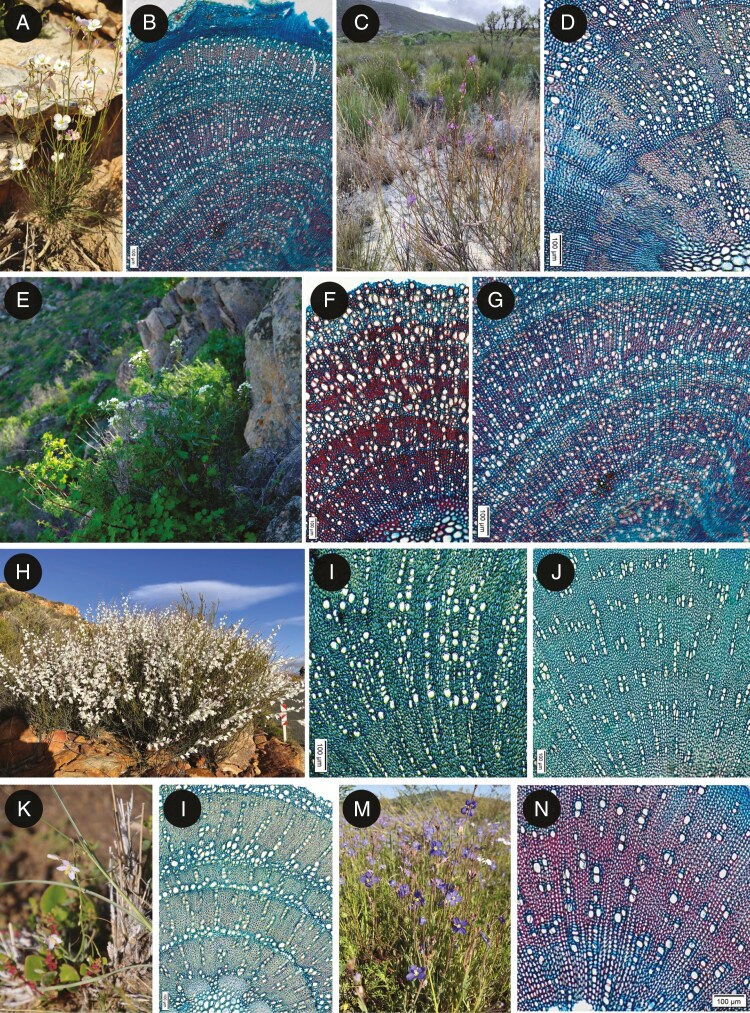
An overview of habit and wood anatomical diversity of *Heliophila*. (A, B) *Heliophila carnosa* is a perennial subshrub with herbaceous shoots. (C, D) Perennial *H. maraisiana*. (E, F) *Heliophila eximia* is perennial and slightly woody. (G) Shrubby *H. carnosa*. (H, I) *Heliophila juncea* grows as a sizeable wiry shrub over 1 m tall. (J) *Heliophila schulzii* is a monocarpic annual. (K, L) *Heliophila minima* is a poorly known herbaceous species. (M, N) *Heliophila africana* is a common annual.

To test whether the evolutionary shifts between annual and perennial lifespans affect selective pressure experienced by wood functional traits and to assess climatic drivers of secondary woodiness, we used *Heliophila* Burm.f. ex L. (Brassicaceae; Fig. 1), one of the hyperdiverse ‘Cape clades’ comprising nearly 100 species (POWO, 2024), which disproportionately contribute to the botanical diversity of the Cape Floristic Region (CFR) of South Africa (Linder, 2003). Its species are either annuals or polycarpic perennials, and the clade has undergone multiple independent lifespan transitions (Monroe *et al.*, 2019). Using distribution data, we first reconstructed climatic niches that select for each lifespan and for secondarily woody habit. We then used a highly curated specimen collection to measure nine wood anatomical traits (Table 1) in almost half of the currently recognized species and resolved the most inclusive phylogeny of *Heliophila* available. We reconstructed evolutionary shifts between lifespans and assessed if wood traits evolve under distinct selective pressures in annual and perennial species by comparing the fit of Brownian motion (BM) and Ornstein–Uhlenbeck (OU) models to our dataset. Next, we examined the evolutionary trait optima (θ), which are estimated trait values towards which species within a given selective regime (annual/perennial) are expected to evolve in the long run if the current selective pressures (identified based on empirical data) remain unchanged. Finally, we used the current climatic niches as predictors of the observed (in contrast to estimated θ) wood traits to assess if *Heliophila* is a case of wood anatomy ‘lagging behind’ lifespan shifts. This is the first evolutionarily informed study to examine the effects of lifespan shifts on wood anatomy.

## MATERIALS AND METHODS

### Data sources

#### Taxonomic identity

In a prior study examining the correlation between lifespan and drought frequency in *Heliophila* (Monroe *et al.*, 2019), oversight in accounting for misidentified herbarium and GenBank accessions resulted in accurate representation for less than 25 % of studied species (Mucina *et al.*, 2024). To mitigate this issue, we exclusively drew samples from the finely curated collection at Compton Herbarium (South African National Biodiversity Institute, SANBI, Cape Town, South Africa), and each plant’s taxonomic identity was confirmed by an expert co-author (P.W.).

#### Sampling

 For wood anatomy, we sampled 68 specimens representing 47 species, including one not yet formally named. Plants were cut at a similar level *c*. 15 cm below the stem tip to mitigate the vessel tapering effect (Rosell *et al*., 2017).

To resolve phylogeny, we selected 127 sequences of nuclear ribosomal ITS1–2 from GenBank, avoiding sequences that were misidentified in previous studies (Mummenhoff *et al.*, 2005; Mandáková *et al.*, 2012; Dogan *et al.*, 2021; Mucina *et al.*, 2024). These were complemented by 52 newly obtained sequences. Molecular sampling encompassed 81 species and seven taxa awaiting formal description or reinstatement, constituting ~84 % of the known specific diversity of *Heliophila* (POWO, 2024). Supplementary Data Table S1 provides unique herbarium identifiers and GenBank IDs for all sequences.

#### Wood anatomy

Standard anatomical procedures were employed to obtain macerated wood, transverse and longitudinal stem sections (Frankiewicz *et al.*, 2022). We measured nine anatomical traits: horizontal and vertical intervessel pit diameters, vessel diameter, density, grouping, wall thickness, vessel element length, fibre length and wall thickness (IAWA Committee, 1989). The means and medians of these traits were calculated for each species. Due to the proximity of the mean and median values, only means were used in the following steps. Supplementary Data Table S1 contains all raw and aggregated measurements.

#### Lifespan and habit

To determine species lifespan (annuals, perennials), we utilized literature data, collectors’ notes on herbarium specimens and over 30 years of field-based observations (P.W.). Six species (*H. brassicifolia*, *H. carnosa*, *H. minima*, *H. pseudoeximia*, *H. rigidiscula*, *H. suavissima*) show interpopulation polymorphisms and can be treated either as annuals or perennials with annual shoots. We conducted downstream analyses twice, changing the assignment of these six species between lifespan categories, but without significant effect on the final results. We then identified secondarily woody species, defined as those producing wood from the base of the stem to its upper parts, following the definition by Kidner *et al*., (2015). Supplementary Data Table S1 contains lifespan and woodiness data.

#### Climatic data

The occurrence dataset was compiled in three steps. We initially retrieved occurrence records for each species from the Global Biodiversity Information Facility (GBIF). GBIF incorporates the Botanical Database of South Africa (BODATSA), curated by SANBI, and non-curated sources. In the second step, we reviewed each record, manually excluding entries with spurious taxonomic identification or incorrect/unlikely coordinates. In the case of species with fewer observations, this was carried out until all records were reviewed (e.g. *H. minima* with 112 records, of which we kept 53). For those overrepresented, we continued until the whole range of the species’ distribution was reflected in our dataset (e.g. *H. carnosa* with 457 records, of which 156 were kept). To ascertain coverage of a species’ entire range, we cross-referenced distribution information from original species descriptions with those generated by plotting records in our datasets (Supplementary Data Fig. S1). We prioritized accuracy over quantity, favouring fewer well-supported observations, particularly those with accompanying photos. After removing duplicates, the finalized dataset comprised 2477 records spanning 68 species. In the following step, we extracted bioclimatic variables for each record from WorldClim (Fick and Hijmans, 2017) at a spatial resolution of 30 s. We aggregated them by the mean to obtain values for each species. We then conducted a principal component analysis (PCA) on the resulting dataset to reduce the original 19 variables to a smaller set of uncorrelated principal components (Table 2). These components were subsequently used as proxies for the climatic niche.

**Table 2.** Loadings of 19 bioclimatic variables on the first three principal components. Variables with major contributions to each component (loading > 0.3) are highlighted in bold. Percentages indicate explained variance.

Bioclimatic variable	PC1 (44.78 %)	PC2 (25.96 %)	PC3 (13.09 %)
BIO1 = Annual Mean Temperature	−0.19	**0.36**	−0.09
BIO2 = Mean Diurnal Range	−0.28	−0.22	−0.01
BIO3 = Isothermality	−0.21	0.13	**−0.32**
BIO4 = Temperature Seasonality	−0.20	**−0.30**	0.18
BIO5 = Max Temperature of Warmest Month	−0.31	0.10	0.07
BIO6 = Min Temperature of Coldest Month	−0.01	**0.44**	−0.02
BIO7 = Temperature Annual Range	−0.24	−0.28	0.07
BIO8 = Mean Temperature of Wettest Quarter	−0.11	0.13	**−0.51**
BIO9 = Mean Temperature of Driest Quarter	−0.09	0.28	**0.41**
BIO10 = Mean Temperature of Warmest Quarter	−0.25	0.26	−0.02
BIO11 = Mean Temperature of Coldest Quarter	−0.10	**0.42**	−0.12
BIO12 = Annual Precipitation	**0.32**	0.07	−0.04
BIO13 = Precipitation of Wettest Month	**0.30**	0.10	0.04
BIO14 = Precipitation of Driest Month	**0.30**	−0.03	−0.09
BIO15 = Precipitation Seasonality	−0.04	0.20	0.25
BIO16 = Precipitation of Wettest Quarter	**0.30**	0.11	0.05
BIO17 = Precipitation of Driest Quarter	**0.30**	−0.02	−0.13
BIO18 = Precipitation of Warmest Quarter	0.18	−0.07	**−0.41**
BIO19 = Precipitation of Coldest Quarter	0.24	0.16	**0.37**

### Analyses

#### Phylogeny

We aligned the sequences using the E-INS-i algorithm in MAFFT 7.271 (Katoh and Standley, 2013) and removed the ambiguously aligned positions with the ‘automated1’ algorithm in trimAL 1.2 (Capella-Gutiérrez *et al.*, 2009). We used PartitionFinder2 to select the best nucleotide substitution model (GTR+G) (Lanfear *et al.*, 2012) and MrBayes with default priors to resolve phylogeny (Ronquist *et al.*, 2012). The analysis was performed in two simultaneous runs for 5 000 000 generations each and sampled every 1000 generations. The convergence of analysis was assessed using Tracer v.1.7.1 (Rambaut *et al.*, 2018). The initial 25 % of trees were discarded as burn-in. To account for phylogenetic uncertainty, the sample of 100 trees from the stationary phase was used for subsequent analyses.

#### Climatic niche, lifespan and habit evolution

In the first step, we tested the predictive capabilities of climatic niche occupation on lifespan in *Heliophila*. This was achieved by employing a phylogenetic extension of a binomial logit model with annuals coded as 0 and perennials as 1. We then applied the same approach to habit (herbs coded as 0, secondarily woody species as 1). The analysis was performed using the ‘phyloglm’ function, as implemented in the R package *phylolm* (Tung Ho and Ané, 2014), with MPLE method of penalized likelihood optimization, and 1000 bootstrap replicates to estimate the coefficients.

#### Ancestral state reconstruction and regime mapping

Reconstruction of the evolutionary history of *Heliophila* lifespan (annual/perennial) and habit (herbaceous/secondarily woody) was conducted with the R package *corHMM* using marginal likelihood with 20 restarts (Boyko and Beaulieu, 2021). We initially tested two models assuming different transition rate matrices, ER (equal rates, i.e. single rate parameter for changes in both directions) and ARD (all rates different, i.e. with two parameters). Since, for both characters, the more complex model (ARD) did not show a significant improvement in terms of fit across sampled trees [ΔAICc (delta Akaike information criterion) < 2], we opted for ER in downstream analyses. The estimated transition rate matrices were used to generate stochastic maps (‘makeSimmap’ function), which were then employed to delimit selective regimes on the sample of trees.

#### Anatomical optima in annuals/perennials

To test for the presence of lifespan-specific regimes in the evolution of *Heliophila* wood anatomy, we performed analyses using the R package *mvMORPH* (Clavel *et al.*, 2015). We fitted four multivariate models: simple Brownian Motion (BM1) – drift with global evolutionary rate parameter (σ); Brownian motion with multiple rates (BMM) – drift with independent evolutionary rate parameters across regimes; simple Ornstein–Uhlenbeck model (OU1) – adaptive evolution with global evolutionary rate parameter, phenotypic optima (θ) and strength of selection (α); and Ornstein–Uhlenbeck model with multiple optima (OUM) – adaptive evolution with global evolutionary rate parameter and strength of selection but distinct phenotypic optima across regimes. The optimizations were performed using the ‘rpf’ method for likelihood calculation, with tree height scaled at unity and root assumed at the oldest regime state stationary distribution (‘root = FALSE’). Best-fitting models were selected based on AICc. We did not conduct a similar analysis for habit-specific regimes (herbaceous/secondarily woody) due to the small number of secondarily woody species.

#### Climate as a predictor of wood traits

In this part, we used the climatic proxies (*PC1–PC3*; from the PCA of bioclimatic variables) as multivariate continuous predictors of the wood anatomical trait values (*anatomy)*. Due to the fact that perennials and annuals may respond differently to changes in climatic niches, we also included interaction terms between each PC and lifespan (*lsp*). Additionally, since we suspect that lifespan may directly affect at least some anatomical traits irrespective of climate, we included it as a covariate in the model alongside interaction terms. The final formula was:


$$\begin{array}{lll} & anatomy\;∼\;PC1\;+\;PC2\;+\;PC3\;+\;lsp\;+\;\left(PC1\;\times \;lsp\right)\; & +\;\left(PC2\;\times \;lsp\right)\;+\;\left(PC3\;\times \;lsp\right) \end{array}$$

To this end, we first fitted two phylogenetic linear models (pGLS) assuming evolution under BM or OU using penalized likelihood with the ‘RidgeArch’ penalty. To avoid potential bias in model selection in favour of the OU process, the measurement error was estimated from each dataset and used as a nuisance parameter during optimization (Silvestro *et al.*, 2015; Clavel *et al.*, 2019). To test for the effect of predictors, best-fitting models chosen based on the Extended Information Criterion were provided as input in type II phylogenetic MANOVA (pMANOVA) and assessed using Pillai’s statistic with 1000 permutations.

## RESULTS

### Evolution of lifespan and habit

**Fig. 2.**
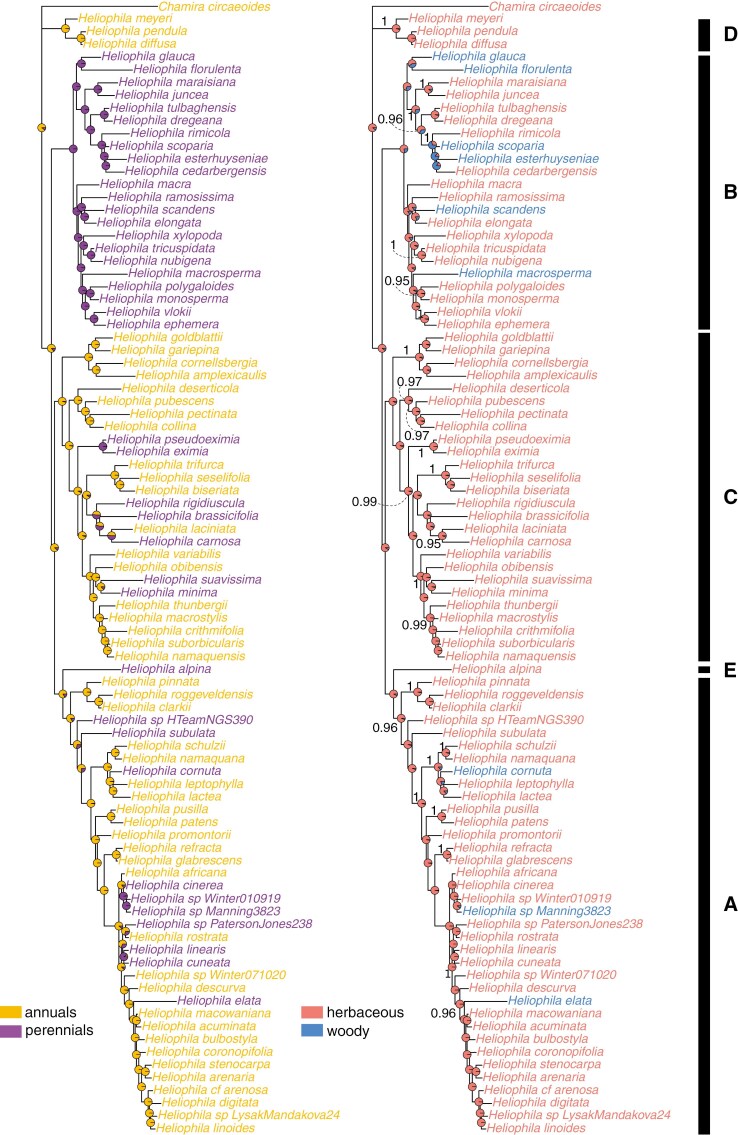
The 50 % majority rule phylogeny of *Heliophila* with ancestral state reconstruction for lifespan (left) and habit (right). Pie charts show the marginal relative likelihood of a given state. Branch support values correspond to a posterior probability (only values ≥0.95 are shown) and are the same for both phylogenies. The main clades are marked A–E, following the convention of previous authors.

Our phylogeny of *Heliophila* is congruent with previous studies and consists of five major clades designated A–E (Mandáková *et al.*, 2012; Dogan *et al.*, 2021). Annual lifespan is plesiomorphic for *Heliophila*; shifts to perennial lifespan occurred at least ten times and were followed by three possible reversals (Fig. 2). Three clades are homogeneous concerning the lifespan: monospecific clade E (*H. alpina*) and much more numerous clade B are perennial, while small clade D consists of annuals. Whereas clade C is predominantly annual, five probable shifts to perennial lifespan were reconstructed. The remaining shifts to perennial lifespan and all reversals were reconstructed in clade A. Four of the ten perennial species in clade A are yet to be formally recognized.

The herbaceous habit was reconstructed as ancestral to *Heliophila* (Fig. 2), with eight out of nine instances of secondary woodiness resulting from independent evolutionary transitions (only *H. scoparia* and *H. esterhuyseniae* share a recent woody ancestor). All secondarily woody species belong either to clade B (six spp.) or A (three spp.).

### Climatic niche of Heliophila

Following the investigation of the scree plot, we retained the first three principal components (Table 2) for downstream analyses, explaining almost 84 % of the variance in the dataset. The first principal component (PC1) accounts for 44.78 % of the variance and represents water availability, with major contributions from five bioclimatic variables: max temperature of the warmest month (BIO5; −0.31), annual precipitation (BIO12; 0.31), precipitation of the wettest month (BIO13; 0.30), precipitation of the driest month (BIO14; 0.30) and precipitation of the wettest quarter (BIO16; 0.30). The second principal component (PC2), accounting for 25.96 % of the variance, stands for overall temperature and is aligned with three climatic parameters: annual mean temperature (BIO1; 0.36), min temperature of the coldest month (BIO6; 0.44) and mean temperature of the coldest quarter (BIO11; 0.42). The third principal component (PC3) can be interpreted as expressing Mediterranean-type seasonality with a strong negative contribution of isothermality (BIO3; −0.32), mean temperature of the wettest quarter (BIO8; −0.51), mean temperature of the driest quarter (BIO9; 0.41), precipitation of the warmest quarter (BIO18; −0.41) and precipitation of the coldest quarter (BIO19; 0.37).

**Fig. 3.**
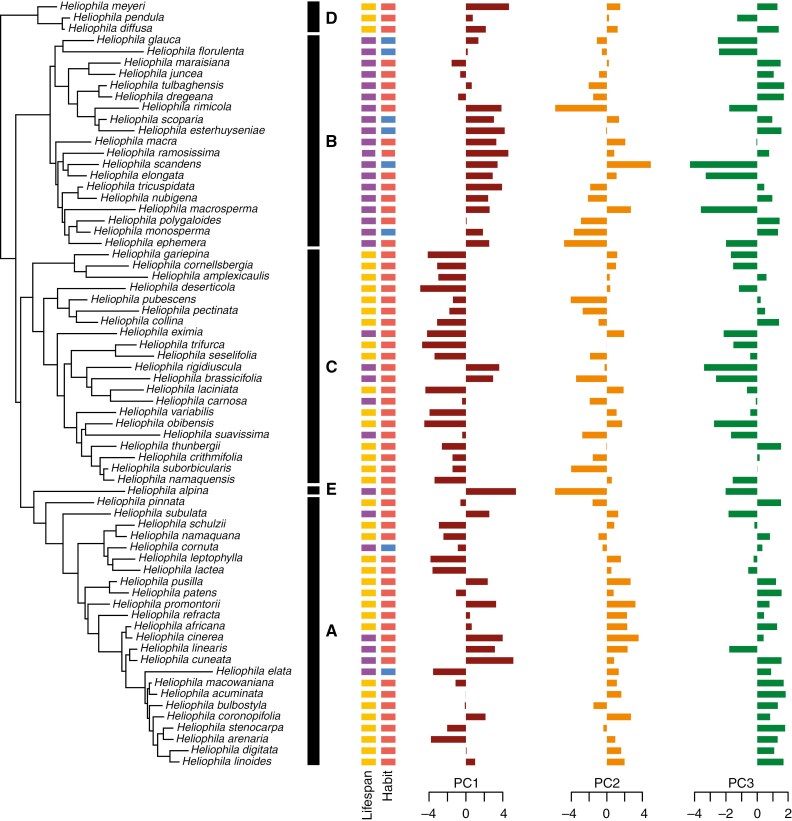
Climatic niche reconstruction in *Heliophila*. Square boxes indicate character states for lifespan and habit. Bars represent values of the first three principal components, describing water availability (PC1), temperature (PC2) and Mediterranean-type seasonality (PC3). The consensus tree was pruned to match 68 species used in PCA.

Taken together, annual species occupy a region of the climatospace with lower PC1 (i.e. driest niches) and higher PC3 (i.e. more pronounced Mediterranean-type seasonality) values (Table 3). This is particularly true for annuals in clade C, while those from clades A and D cluster near the centre of the climatospace, exhibiting relatively higher values for all three principal components. This pattern (i.e. lower PC1 and higher PC3 values in annuals than perennials) is retained when considered on a clade-wise basis (Fig. 3). Perennials are distributed between two distinct groups with either less negative (those from clades A and B) or more negative (those from clades C and E) PC3 values. The PC2 values for all annuals are higher (i.e. warmer niches) than for perennials. However, within each clade, both lifespans have more similar PC2 values, so the genus-wise differences may be driven by outliers (e.g. monospecific clade E that contains perennial *H. alpina*). At the same time, we were unable to identify a clear pattern of climatospace occupation for secondarily woody species of *Heliophila*.

**Table 3.** Climatic proxy values from the principal component analysis for all annual and perennial species and separately for each clade. Lifespans in clades B, D and E are homogenous; clade E is monospecific.

Clade/climatic proxy	PC1		PC2		PC3	
	Annuals	Perennials	Annuals	Perennials	Annuals	Perennials
*Heliophila*	−1.5 ± 2.5	1.8 ± 2.5	0.4 ± 1.8	−0.6 ± 2.6	0.3 ± 1.2	−0.6 ± 1.9
Clade A	−0.6 ± 2.2	1.7 ± 3.3	1.1 ± 1.4	1.5 ± 1.4	1.0 ± 0.7	−0.1 ± 1.4
Clade B	–	2.0 ± 1.9	–	−0.8 ± 2.6	–	−0.3 ± 2.1
Clade C	−3.2 ± 1.2	0.3 ± 3.1	−0.4 ± 1.9	−1.3 ± 2.2	−0.5 ± 1.2	−2.0 ± 1.2
Clade D	2.5 ± 2.0	–	1.0 ± 0.7	–	0.5 ± 1.5	–
Clade E	–	5.4	–	−5.8	–	−2.0

### Climate as a predictor of lifespan and secondary woodiness

**Fig. 4.**
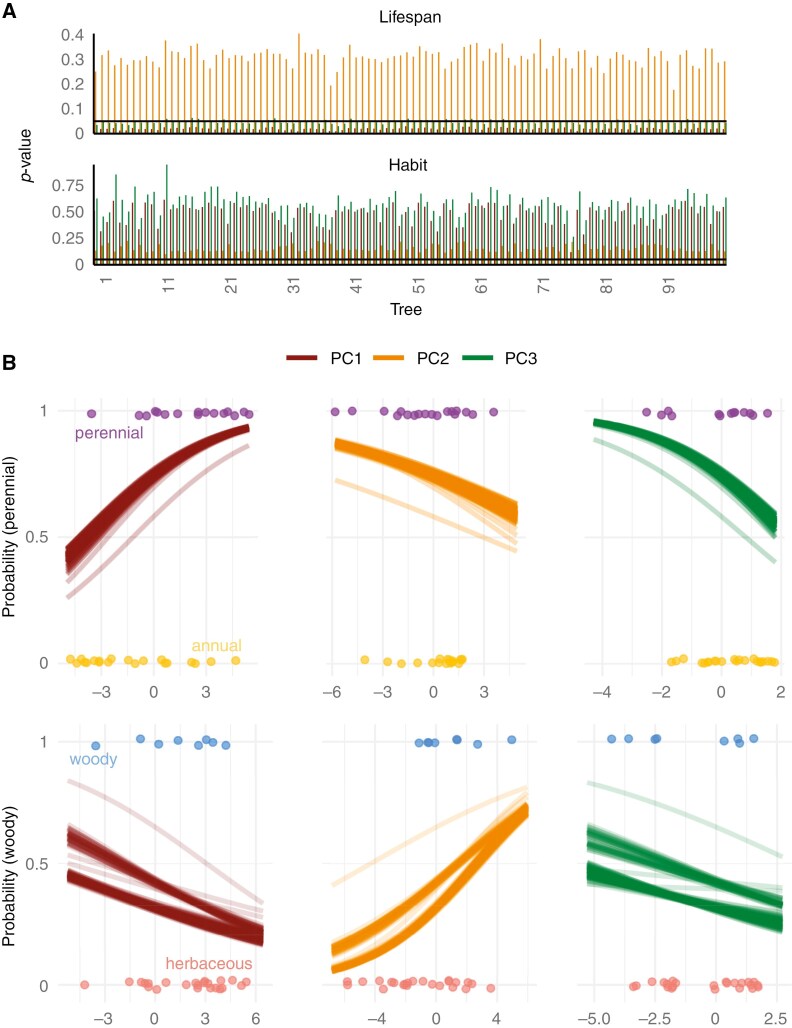
Phylogenetic logistic regression predicting the probability of a perennial habit as a function of climatic niche occupation, optimized across 100 randomly sampled trees. (A) *P*-values for the effect of each climatic PC on lifespan (upper) and habit (lower), with the black line indicating the 0.05 significance threshold; (B) regression curves for each PC and the distribution of either annuals and perennials (upper) or herbaceous and secondarily woody species (lower) along the principal component axes.

The results of phylogenetic logistic regression support the association between climate and lifespan in *Heliophila* (Fig. 4). High water availability (PC1) was identified as a significant predictor of perennial lifespan (*P* < 0.05) across all 100 trees analysed, consistent with the patterns observed in the PCA (Table 3; Fig. 3). Perennial lifespan also showed a significant correlation with lower values of PC3 in 70 trees. The association between temperature (PC2) and evolutionary lifespan shifts in *Heliophila* was insignificant.

Regardless of phylogeny, none of the climatic principal components emerged as a significant predictor of secondary woodiness in *Heliophila*. Among the three analysed PCs, the relationship between woodiness and temperature was the closest to the significance threshold. Secondarily woody species appeared to occupy higher PC2 values than their herbaceous relatives (Fig. 4), but the small sample size limits the strength of this inference.

### Evolutionary wood trait optima of annual/perennial species

**Fig. 5.**
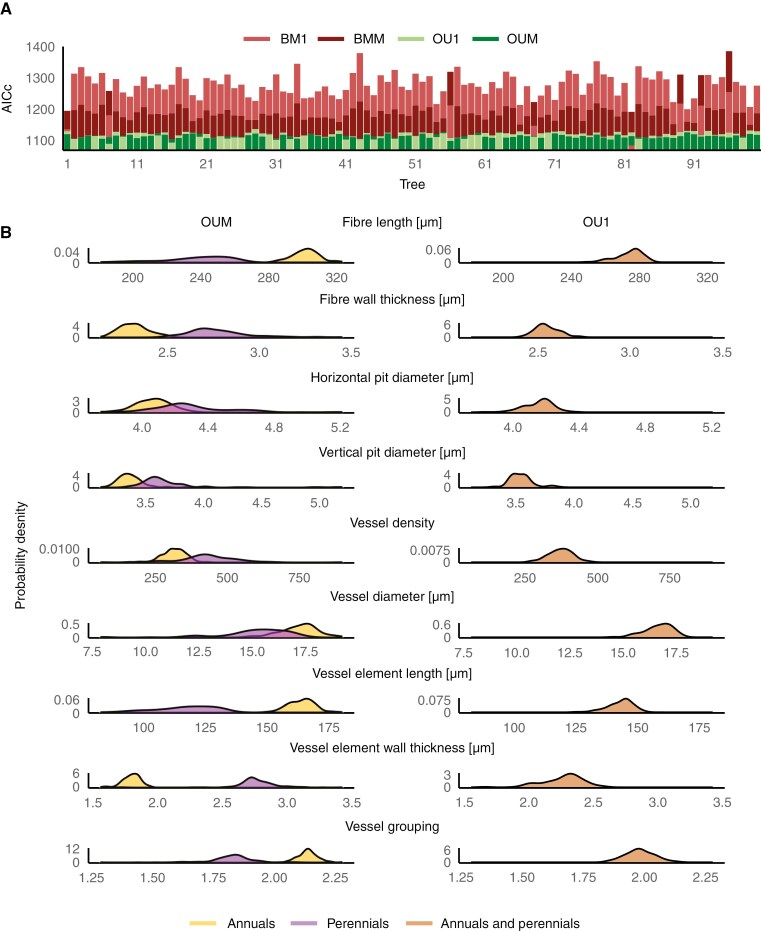
Multivariate model fitting to test for the presence of lifespan-specific optima in *Heliophila* wood anatomy. The upper panel (A) illustrates cumulative AICc plots for models, optimized across 100 randomly sampled trees. The lower panels (B) show probability density plots for the optima estimated under the OUM and OU1 models.

Our analyses provide robust support for adaptive models across 99 out of 100 trees. However, they remain largely inconclusive regarding the distinction between single versus multiple optima scenarios. In reconstructions where a single model was preferred, the OU1 model was favoured over the OUM model (51 trees vs. 37 trees). For 12 trees, both models exhibited a similar fit. The optima estimated under the OUM model (Fig. 5) show considerable overlap between annuals and perennials for four traits, while traits related to the mechanical resistance of stems – fibre length (302 ± 8 vs. 238 ± 20 µm), vessel element length (164 ± 6 vs. 117 ± 12 µm), fibre wall thickness (2.32 ± 0.08 vs. 2.78 ± 0.17 µm), vessel element wall thickness (1.79 ± 0.07 vs. 2.76 ± 0.19 µm) – as well as vessel grouping (2.14 ± 0.04 vs. 1.81 ± 0.11) have mostly a disjoint distribution. When the traits with a disjoint distribution were analysed independently from the remaining traits, they consistently supported the OUM model (Supplementary Data Fig. S2). In the simpler OU1 model, the recovered optima for traits tend to fall near the average of the two regime-specific optima estimated under the OUM model.

Surprisingly, while these mechanical traits are probably subject to distinct selective pressures in annuals and perennials, they also display the highest values for phylogenetic half-lives (the time it takes for a trait to move halfway from its ancestral state to a new optimum; Supplementary Data Table S2). Given that annual habit was ancestral in *Heliophila*, this may suggest that mechanical-support traits and vessel grouping exhibit significant phylogenetic inertia, i.e. these traits retain ancestral-like characteristics for longer periods despite exposure to new selective pressures.

### Climate as a predictor of observed wood traits

Our analyses were inconclusive in determining the evolutionary model that best describes the relationship between climatic niche occupation and wood anatomical traits. The BM model was the best fit for 57 trees, while the OU model was favoured for 24 trees. For 19 trees, both models performed similarly (ΔAICc < 2). Although there was no clear consensus on which model best explains the data, the estimated coefficients and the results of the pMANOVA (Table 4) were consistent across all trees, regardless of whether the BM or OU model was preferred. Among all analysed predictors, only the effect of lifespan was unanimously significant at *P*-value cut-off of 0.05. This indicates that climate is not a good predictor of wood anatomy in *Heliophila*, irrespective of annual/perennial habit. The estimated odds ratios for the association between individual traits and perennial habit align well with the results of the OU1/OUM model fitting. Perennials are significantly more likely to have short (odds ratio = 0.22 ± 0.005, based on 100 trees and BM model), thick-walled vessel elements (3.79 ± 0.002), high vessel density (2.48 ± 0.004), and short (0.40 ± 0.002) but thick-walled (1.33 ± 0.003) fibres. Additionally, perennials are less likely to show high vessel grouping (0.82 ± 0.009) and diameter (0.79 ± 0.007), as well as high vertical (0.73 ± 0.01) and horizontal (0.89 ± 0.003) pit diameters.

**Table 4.** Summary of multivariate linear model fit for the association between wood anatomy and climate, presented as means and standard deviations of *P*-values and coefficients estimated from 100 randomly sampled trees.

Response/predictor	PC1 (annual)	PC2 (annual)	PC3 (annual)	PC1 (perennial)	PC2 (perennial)	PC3 (perennial)	Lifespan (perennial)
**BM**							
*P*-value	0.465 ± 0.022	0.52 ± 0.027	0.84 ± 0.034	0.74 ± 0.022	0.116 ± 0.013	0.725 ± 0.031	0.007 ± 0.003
Horizontal pit diameter (µm)	0.109 ± 0.003	−0.215 ± 0.011	−0.032 ± 0.009	−0.104 ± 0.001	0.218 ± 0.006	−0.214 ± 0.005	−0.118 ± 0.003
Vertical pit diameter (µm)	0.135 ± 0.001	−0.432 ± 0.002	−0.222 ± 0.002	−0.16 ± 0.003	0.551 ± 0.001	−0.041 ± 0.003	−0.312 ± 0.01
Vessel diameter (µm)	−0.008 ± 0.001	−0.192 ± 0.008	−0.175 ± 0.003	0.046 ± 0.002	0.438 ± 0.004	0.285 ± 0.004	−0.232 ± 0.007
Vessel density	−0.031 ± 0.004	0.034 ± 0.007	0.056 ± 0.005	−0.181 ± 0.003	−0.144 ± 0.005	0.019 ± 0.001	0.909 ± 0.004
Vessel grouping mean	−0.147 ± 0.002	−0.369 ± 0.001	0.089 ± 0.008	0.106 ± 0.002	0.599 ± 0.002	−0.078 ± 0.005	−0.201 ± 0.009
Vessel element length (µm)	0.235 ± 0.001	−0.048 ± 0.01	−0.143 ± 0.007	−0.041 ± 0.001	0.282 ± 0.006	0.227 ± 0.006	−1.502 ± 0.005
Vessel element wall thickness (µm)	−0.02 ± 0.001	−0.162 ± 0.001	−0.16 ± 0.004	−0.073 ± 0.001	0.233 ± 0.001	0.156 ± 0.001	1.332 ± 0.002
Fibre length (µm)	0.058 ± 0.003	−0.032 ± 0.015	0.07 ± 0.011	0.108 ± 0.002	0.319 ± 0.011	−0.04 ± 0.005	−0.907 ± 0.002
Fibre wall thickness (µm)	0.043 ± 0.001	−0.212 ± 0.002	−0.39 ± 0.005	−0.009 ± 0.002	0.278 ± 0.001	0.437 ± 0.004	0.286 ± 0.003
**OU**							
*P*-value	0.45 ± 0.05	0.514 ± 0.033	0.832 ± 0.046	0.728 ± 0.042	0.109 ± 0.022	0.712 ± 0.041	0.006 ± 0.002
Horizontal pit diameter (µm)	0.109 ± 0.004	−0.211 ± 0.02	−0.035 ± 0.007	−0.103 ± 0.002	0.215 ± 0.014	−0.212 ± 0.005	−0.115 ± 0.01
Vertical pit diameter (µm)	0.134 ± 0.003	−0.438 ± 0.017	−0.222 ± 0.005	−0.158 ± 0.007	0.558 ± 0.016	−0.039 ± 0.007	−0.312 ± 0.015
Vessel diameter (µm)	−0.01 ± 0.004	−0.203 ± 0.03	−0.179 ± 0.01	0.047 ± 0.003	0.446 ± 0.021	0.288 ± 0.011	−0.233 ± 0.008
Vessel density	−0.03 ± 0.006	0.038 ± 0.015	0.056 ± 0.006	−0.18 ± 0.003	−0.146 ± 0.011	0.019 ± 0.002	0.906 ± 0.008
Vessel grouping mean	−0.146 ± 0.002	−0.364 ± 0.012	0.088 ± 0.012	0.106 ± 0.002	0.596 ± 0.01	−0.076 ± 0.01	−0.198 ± 0.016
Vessel element length (µm)	0.235 ± 0.002	−0.059 ± 0.033	−0.147 ± 0.016	−0.043 ± 0.004	0.291 ± 0.025	0.23 ± 0.013	−1.508 ± 0.011
Vessel element wall thickness (µm)	−0.021 ± 0.001	−0.167 ± 0.01	−0.161 ± 0.003	−0.074 ± 0.003	0.237 ± 0.01	0.157 ± 0.002	1.331 ± 0.005
Fibre length (µm)	0.056 ± 0.006	−0.044 ± 0.038	0.066 ± 0.019	0.108 ± 0.003	0.329 ± 0.032	−0.037 ± 0.01	−0.907 ± 0.004
Fibre wall thickness (µm)	0.043 ± 0.003	−0.214 ± 0.006	−0.391 ± 0.008	−0.011 ± 0.005	0.281 ± 0.009	0.437 ± 0.008	0.284 ± 0.011

## DISCUSSION

### Water availability drives lifespan shifts

Water availability, rather than temperature, plays the primary role in driving evolutionary transitions between lifespans in *Heliophila*. In this genus, perennials tend to be selected in wetter environments (Fig. 4), as indicated not only by the reconstructed transitions but also by the climatic niche preferences of extant species (Fig. 3). Another important factor influencing lifespan shifts in *Heliophila* is Mediterranean-type seasonality, characterized by hot, dry summers and mild, wet winters. In these habitats, annuals are favoured, whereas perennials are more likely to evolve in environments with less pronounced seasonality (Fig. 4). However, the climatic niches of extant species do not strictly follow these trends, with approximately half of the studied perennials inhabiting niches that are similar to those of annuals concerning the degree of seasonality (Fig. 3). We suspect that this similarity may be superficial, with extant perennials, in fact, occupying less seasonal microhabitats that allow for utilization of fog and dew in addition to rain – a distinction unlikely to be picked up in a large-scale approach. All in all, our results align with Monroe *et al.* (2019), who concluded that the annual lifespan in *Heliophila* evolved as an adaptation to avoid predictable dry seasons.

### Temperature may influence secondary woodiness

The only global analysis of secondary woodiness evolution so far (Zizka *et al.*, 2022) found that on oceanic islands, its occurrence is best explained by favourable aseasonal climate – characterized by climate stability over geological time and the absence or low frequency of frost days – followed by drought-related factors, including higher precipitation seasonality and lower summer rainfall. Additionally, competition-related factors, such as island isolation, play a role. When continental shelf islands were included, herbivory (or rather, its absence) became the strongest predictor. Drought remained important, with a lower aridity index (indicating drier conditions) and higher precipitation seasonality correlating with a higher number of secondarily woody species. Additionally, lower temperature seasonality emerged as a factor favouring secondary woodiness. In contrast, mainland secondarily woody species are less understood. For example, *Impatiens* (Lens *et al.*, 2012) and Southern African umbellifers (Frankiewicz *et al.*, 2021) show both woody and herbaceous forms in similar niches, while woody *Begonia* are found primarily on tropical mountain slopes, where they are sheltered from drought (Kidner *et al.*, 2015).

Our study showed that lifespan extension – a prerequisite for the evolution of secondary woodiness – occurs in *Heliophila* within wetter and less seasonal niches, consistent with the predictions of the favourable aseasonal climate hypothesis (Carlquist, 1974). However, among the derived perennial species, only nine develop wood from the base to the upper twigs and can, therefore, be considered secondarily woody (Fig. 2). Our data suggest that secondary woodiness in *Heliophila* may be more likely to evolve in niches with higher overall temperatures (represented by PC2 in our analysis; Fig. 4). However, this trend is not statistically supported, probably due to the limited number of transitions from herbaceous to woody life forms. Nevertheless, the observation is intriguing given the potential link between higher temperatures and increased lignification of wood (Crivellaro *et al.*, 2022; Büntgen *et al.*, 2023; Körner *et al.*, 2023). Further studies are essential, as the drivers of secondary woodiness probably differ between islands and the mainland, as well as between clades.

### Lifespans select for distinct wood trait optima

Our study confirms that annual and perennial species experience distinct selective pressures on wood functional traits related to the mechanical resistance of the stem and vessel grouping. These differing selective pressures on stem mechanical traits probably result from the fact that perennials reuse their stems across multiple growing seasons, making denser and more robust wood advantageous (Kallarackal and Ramírez, 2024). In contrast, annuals favour traits that support rapid growth and which are less costly and quicker to produce. Specifically, perennials are selected for fibre and vessel element walls that are 20–55 % thicker than annuals (Fig. 5; Supplementary Data Table S2), reinforcing the stems. Annuals, on the other hand, have thinner cell walls, but their fibres and vessel elements are 30–40 % longer than those in perennials. The correlation between fibre and vessel element length is expected, as they originate from the same fusiform cambial initials. Longer fibres could potentially enhance the mechanical strength of the stem by increasing the contact area with surrounding cells, which might compensate for the thinner fibre walls found in annuals. Nevertheless, this remains a hypothesis, as experimental evidence is currently lacking, and it cannot be ruled out that the differences are non-adaptive. We also retrieved distinct selective pressures on vessel grouping optima, with more grouped vessels reconstructed in the evolution of annuals. This, however, may be non-adaptive, as it is often observed that vessels are more clustered in first-produced wood due to limited space for their distribution.

### Wood anatomy lags behind lifespan shifts

Although the correlation between wood anatomy and climate is well-documented across various clades and globally (e.g. Carlquist, 1966, 2001; Carlquist and Hoekman, 1985; Jansen *et al.*, 2004; Beeckman, 2016; Frankiewicz *et al.*, 2020a, 2020b), in *Heliophila*, climatic proxies did not effectively predict wood trait values, whereas lifespan proved to be a strong predictor (Table 4). There are two possible explanations for this. First, using annual means to calculate climatic niche proxies might overlook the fact that plants are shaped by the conditions they experience during active growth, not dormancy. This may be less of an issue for perennials, which grow for most of the year, but could introduce bias for annuals in highly seasonal habitats. However, our estimates of the growing season (Supplementary Data Table S1) show that most annuals grow actively for 7–8 months, reducing the impact of using annual means. Moreover, we addressed this by including an interaction term between lifespan and climatic proxies, which allowed us to account for differences in the response of wood anatomy to climate between annuals and perennials. We expected climate to better predict wood traits in perennials, but this was not supported – climate had no significant effect on wood anatomy in *Heliophila*, regardless of lifespan.

The second explanation for the lack of correlation between wood anatomy and climate could be that wood anatomy lags behind changes in lifespan. We demonstrated that climate, specifically water availability and seasonality, is a key driver of evolutionary shifts in lifespan in *Heliophila*. It is likely that these shifts are recent; depending on the calibration method, previous studies estimated the crown age of *Heliophila* at 1.0–4.4 Mya (Mummenhoff *et al.*, 2005; Verboom *et al.*, 2009; Mandáková *et al.*, 2012), with a more recent study pushing it back to 18 Mya (Dogan *et al.*, 2021). In the case that the younger age is supported, it is likely that some species that have recently transitioned from annual to perennial due to climate drivers may still have wood anatomies similar to annuals rather than to other perennials in similar environments. The same applies to reversals to an annual lifespan. An older age estimate for *Heliophila* does not preclude our interpretation, simply indicating that the five traits selected for distinct optima in annual and perennial lifespans experience weak selection and require long periods to arrive at the new optimum. This is supported by the fact that these five traits are the same traits with the longest half-lives – meaning the time needed for a trait to move halfway from its ancestral state to the new optimum. These traits evolve, on average, 20 times more slowly than the other traits we studied (Supplementary Data Table S2). Thus, we conclude that climate changes first influence lifespan, and adjustments in wood anatomy follow slowly, only reaching their optima over a longer period.

Our interpretation aligns with the *vascular adaptation hypothesis* (Aloni, 2021), which proposes that the environment controls the characteristics of the vascular system by regulating the duration of a plant’s active growth, subsequently affecting its height and shape. The plant’s height and shape then determine the hormonal gradients that directly regulate wood anatomy. Therefore, according to the hypothesis and in congruence with our interpretation, the lifespan is the first to respond to changing climates, followed by adjustments in stem morphology (not studied here) and only then by wood anatomy. Alongside our findings that adjustments in wood anatomy are slow, this highlights that although plants can adapt to changing climates, the key factor in determining the success of their adaptation is the rate at which climatic changes occur.

## CONCLUSIONS

Our study on *Heliophila* showed that shifts between annual and perennial lifespans are driven primarily by water availability and seasonality, with perennials selected in wetter and less seasonal niches than annuals. Among perennials, we identified nine secondarily woody species, and our data suggest that secondary woodiness may be promoted by warmer niches, but this potential trend was not statistically supported. On the other hand, we showed that annuals and perennials experience distinct selective pressures on wood anatomical traits, particularly those related to stem mechanics. Specifically, annuals are selected for longer wood cells with thinner cell walls, probably providing less mechanical support but allowing faster growth rates. Despite a link between climate and evolutionary lifespan shifts, and between lifespan and wood traits, climate is not an effective predictor of wood trait values. This disconnect between climate and wood anatomy is a case of phylogenetic inertia, where lifespans respond first to climatic pressures and wood anatomy adjusts slowly after a lifespan shift. Our findings align with known mechanisms of hormonal control of wood development and emphasize that while plants can adapt to new environmental conditions, the success of their adaptation is determined by the rate of environmental change.

## SUPPLEMENTARY DATA

Supplementary data are available at *Annals of Botany* online and consist of the following.

**Fig. S1**. Maps with *Heliophila* specimen occurrences grouped by species. **Fig. S2**. Results of model fitting (a) and evolutionary optima under the OUM model (b) for the subset of traits related to mechanical resistance of stem and vessel grouping. **Table S1**. Accession list with specimen IDs, GenBank identifiers, lifespan, vegetative growth season estimates, wood anatomy and occurrence data. **Table S2**. Means and standard deviations for the evolutionary optima and half-lives of wood anatomical traits, estimated under the OU1 and OUM models.

mcaf046_suppl_Supplementary_Materials

## Data Availability

All DNA sequences generated were uploaded to GenBank, and their identifiers are given in Table S1. The code used for the analyses is available from J.B. GitHub (https://github.com/JaJeBacz/Heliophila).
