# Supplementary material for: Lifespan outperforms climate as a predictor of wood functional traits, but secondary woodiness shows no clear climatic pattern in Heliophila, a diverse clade from the Cape Floristic Region
Source: Ann Bot. 2025 Mar 19;138(3):793–806. doi: 10.1093/aob/mcaf046 (PMC13487432; doi:10.1093/aob/mcaf046)
Supplement: Supplementary file 1 [file mcaf046_suppl_supplementary_materials.zip › mcaf046_suppl_Supplementary_Materials.odt]

Annals of Botany Supporting Information

Article title: Lifespan outperforms climate as a predictor of wood functional traits, but secondary woodiness shows no clear climatic pattern in Heliophila, a diverse clade from the Cape Floristic Region
Authors: J. Baczyński, A.A. Oskolski , P. Winter, R. Manuel, T. Lyner, A.R. Magee, A.M. Muasya, K.E. Frankiewicz

Article acceptance date: ……………….

The following Supporting Information is available for this article:
Figure S1 Maps with Heliophila specimen occurrences grouped by species [available as a separate file from the publisher’s website].
Figure S2 Results of model fitting (a) and evolutionary optima under OUM model (b) for the subset of traits related to mechanical resistance of stem and vessel grouping.
Table S1 Accession list with specimen IDs, GenBank identifiers, lifespan, vegetative growth season estimates, wood anatomy, and occurrence data [available as a separate file from the publisher’s website].
Table S2 Means and standard deviations for the evolutionary optima and half-lives of wood anatomical traits, estimated under the OU1 and OUM models.

Fig. S2 Results of model fitting (a) and evolutionary optima under OUM model (b) for the subset of traits related to mechanical resistance of stem and vessel grouping.

Table S2 Means and standard deviations for the evolutionary optima and half-lives of wood anatomical traits, estimated under the OU1 and OUM models.
Wood trait
Optima
Half-lives

OU1
OUM
(annual)
OUM
(perennial)
OU1
OUM
fibre length [µm]
274 ± 8
302 ± 8
238 ± 20
0.27 ± 0.079
0.16 ± 0.041
fibre wall thickness [µm]
2.55 ± 0.07
2.32 ± 0.08
2.78 ± 0.18
0.51 ± 0.22
0.37 ± 0.18
horizontal pit diameter [µm]
4.16 ± 0.09
4.07 ± 0.11
4.30 ± 0.26
0.0024 ± 0.0014
0.0015 ± 0.0011
vertical pit diameter [µm]
3.54 ± 0.10
3.37 ± 0.11
3.7 ± 0.33
0.0052 ± 0.0021
0.0038 ± 0.0022
vessel density
370 ± 42
314 ± 43
432 ± 108
0.017 ± 0.0052
0.013 ± 0.0052
vessel diameter [µm]
16.7 ± 0.7
17.3 ± 0.9
15.1 ± 1.8
0.0096 ± 0.0042
0.0071 ± 0.0033
vessel element length[µm]
143 ± 5
164 ± 5
117 ± 12
0.066 ± 0.023
0.043 ± 0.015
vessel element wall thickness [µm]
2.27 ± 0.16
1.79 ± 0.07
2.76 ± 0.19
0.13 ± 0.038
0.093 ± 0.026
vessel grouping mean
1.98 ± 0.07
2.14 ± 0.04
1.81 ± 0.11
0.028 ± 0.011
0.022 ± 0.0074
